# An examination of cancer-related fatigue through proposed diagnostic criteria in a sample of cancer patients in Taiwan

**DOI:** 10.1186/1471-2407-11-387

**Published:** 2011-09-06

**Authors:** En-Tien Yeh, Shu-Chuen Lau, Wei-Ju Su, Duu-Jian Tsai, Ying-Yueh Tu, Yuen-Liang Lai

**Affiliations:** 1 Department of Family Medicine, Cardinal Tien Hospital, 362 Zhongzheng Rd, Xindian District, New Taipei City, 231 Taiwan; 2 Department of Hospice and Palliative Care, Cardinal Tien Hospital, 362 Zhongzheng Rd, Xindian District, New Taipei City, 231 Taiwan; 3 School of Medicine, Fu Jen Catholic University, 510 Zhongzheng Rd, Xinzhuang District, New Taipei City, 242 Taiwan; 4 Graduate Institute of Humanities in Medicine, Taipei Medical University, 250 Wu-Hsing Street, Taipei, 110 Taiwan; 5 School of Medicine, Taipei Medical University, 250 Wu-Hsing Street, Taipei, 110 Taiwan; 6 Department of Family Medicine, Taipei Medical University-Shuang Ho Hospital, 291 Jhongjheng Rd, Jhonghe District, New Taipei City, 235 Taiwan; 7 Department of Radiation Oncology, Taipei Medical University-Shuang Ho Hospital, 291 Jhongjheng Rd, Jhonghe District, New Taipei City, 235 Taiwan; 8 Education Center for Humanities and Social Sciences, National Yang-Ming University, 155 Linong Street, Sec 2, Taipei, 112 Taiwan; 9 Mackay Medical College, 46 Zhongzheng Rd, Sec 3, Sanzhi District, New Taipei City, 252 Taiwan

**Keywords:** fatigue, prevalence, diagnosis, cancer

## Abstract

**Background:**

Fatigue among cancer patients has often been reported in the literature; however, great variations have been documented, ranging from 15% to 90%, probably due to the lack of a widely accepted definition and established diagnostic criteria for cancer-related fatigue. The objective of this study was to evaluate the proposed International Statistical Classification of Diseases and Related Health Problems (10^th ^revision) (ICD-10) criteria in a sample of cancer patients from a medical center and a regional teaching hospital in northern Taiwan. More accurate prevalence estimates of CRF may result in improved diagnoses and management of one of the most common symptoms associated with cancer and its treatment.

**Methods:**

Since self-reporting from patients is the most effective and efficient method to measure fatigue, the ICD-10 criteria for fatigue were used. The ICD-10 criteria questionnaire was translated into Chinese and was approved by experts. Patients were recruited from outpatient palliative and oncology clinics and from palliative and oncology inpatient units.

**Results:**

Of the 265 cancer patients that were interviewed between 21 October 2008 and 28 October 2009, 228 (86%) reported having at least 2 weeks of fatigue in the past month, and further evaluation with the ICD-10 criteria showed that 132 (49.8%) had cancer-related fatigue. Internal consistency was very good, which was indicated by a Cronbach alpha of 0.843.

**Conclusion:**

The prevalence of diagnosable CRF in the patients in this sample, of whom most were under palliative treatment, was 49.8%, which was probably somewhat lower than in some of the previous reports that have used less-strict criteria. In addition, among the various criteria of the proposed diagnostic criteria, the most frequently reported symptoms in our sample populations were regarding sleep disturbance and physical factors. Although they will require further replication in other samples, these formal diagnostic criteria can serve as a step toward a common language and a better understanding of the severity range of CRF.

## Background

Fatigue is the most common symptom or complaint related to cancer and cancer therapy [1], and has been identified as the most distressful symptom of cancer patients [2]. Cancer-related fatigue (CRF) profoundly affects the quality of life (QOL) of both the patients and their families, physically and psychosocially, as well as in economic and occupational areas [3].

Cancer-associated fatigue is defined as a subjective state of overwhelming sustained exhaustion and decreased capacity for physical and mental work, which is not relieved by rest [4]. CRF differs from the fatigue that accompanies everyday life, which is usually temporary and relieved by rest. In addition to the direct impact of cancer, various treatment modalities, particularly chemotherapy and radiation, are known to cause fatigue for many patients for an extended period of time. Fatigue caused by cancer or cancer therapy had been proposed for recognition by the World Health Organization (WHO) and for publication in the International Statistical Classification of Diseases and Related Health Problems (10^th ^revision) (ICD-10) [5]. Previous studies have varied in their reports of the prevalence of fatigue in cancer patients. Although not based on formal diagnostic criteria, between 58% and 90% of patients receiving cancer therapy have reported experiencing fatigue [6,7]. The reported prevalence of fatigue in patients with advanced cancer has ranged from 51% to 89% [8,9]. Typically, a cancer patient need only say that he or she is experiencing fatigue to be considered to have CRF. Therefore, existing prevalence estimates may be high and non-discriminating. Current prevalence estimates may combine both patients with everyday fatigue and those who have more clinically significant, definable CRF.

The assessment of fatigue is multidimensional in nature [10]. Ambiguity in the literature and a previous lack of specific tools to measure fatigue created difficulties in establishing assessment and management guidelines. Most practitioners would agree that a working set of diagnostic criteria is essential for research and treatment planning. To end that, the Fatigue Coalition, a multidisciplinary group of medical practitioners, researchers, and patient advocates, proposed a set of diagnostic criteria in 1998 (Table 1). To date, there are no prevalence data in Taiwan using the proposed ICD-10 criteria. The purpose of this study was to examine the prevalence and functional impact of CRF in a sample of Taiwanese cancer patients.

**Table 1:** Proposed (1998 draft) ICD-10 Criteria for Cancer-Related Fatigue

**Six (or more) of the following symptoms have been present every day or nearly every day during the same 2-week period in the past month, and at least one of the symptoms is (A1) significant fatigue**.
A1. Significant fatigue, diminished energy, or increased need to rest, disproportionate to any recent change in activity level
A2. Complaints of generalized weakness or limb heaviness
A3. Diminished concentration or attention
A4. Decreased motivation or interest to engage in usual activities
A5. Insomnia or hypersomnia
A6. Experience of sleep as unrefreshing or nonrestorative
A7. Perceived need to struggle to overcome inactivity
A8. Marked emotional reactivity (eg, sadness, frustration, or irritability) to feeling fatigued
A9. Difficulty completing daily tasks attributed to feeling fatigued
A10. Perceived problems with short-term memory
A11. Postexertional malaise lasting several hours

B. The symptoms cause clinically significant distress or impairment in social, occupational, or other important areas of functioning

C. There is evidence from the history, physical examination, or laboratory findings that the symptoms are a consequence of cancer or cancer therapy.

D. The symptoms are not primarily a consequence of comorbid psychiatric disorders such as major depression, somatization disorder, somatoform disorder, or delirium.

## Methods

### Participants and Settings

Data about cancer patients suspected to be fatigued were recruited from outpatient oncology and palliative clinics and oncology and palliative inpatient units at a medical center and a district teaching hospital in northern Taiwan. Selection criteria required that patients have a pathological diagnosis of cancer, be at least 18 years old, and be able to communicate in Mandarin or Taiwanese. Patients were excluded if they were cognitively impaired, if they refused to participate, or if they could not understand the intent of this study. A final sample of 265 patients was recruited for this study. Fatigue was assessed using the proposed ICD-10 criteria for cancer-related fatigue. Other data collected included demographics (sex, age), educational level, history of disease (type of tumor), staging of disease, and current treatment (e.g., curative treatment, palliative treatment, chemotherapy, radiotherapy, etc.). Written consent was given before the assessment of CRF.

This study was reviewed and approved by the Institutional Review Board of Mackay Memorial Hospital and Cardinal Tien Hospital.

### Instruments

The International Classification of Diseases recently included diagnostic nomenclature for CRF. These criteria have been proposed as a draft of a structured interview guide to help ensure the diagnosis of CRF, and were made in a standardized way across research and clinical applications by Dr. Cella [11]. In 2005, several researchers validated the use of the proposed ICD-10 criteria for fatigue through comparison with other scales, namely the FACT-F and VAS, and discovered that the internal consistency of P-ICD 10 criteria was very good, with an alpha coefficient of 0.82, and concluded that the ICD-10 criteria can be recommended as a diagnostic tool for cancer-related fatigue [12]. We were authorized by Dr. Cella to use the diagnostic interview guide for CRF, and its Chinese-language version, for application in a clinical and research setting to determine the prevalence of CRF in Taiwan.

The English version of the diagnostic interview guide questionnaire was initially translated into Chinese by one of the professional translator, who is bilingual in Chinese and English and experienced in the study topic. A second qualified and blinded bilingual expert back-translated the Chinese version of the diagnostic interview guide questionnaire, creating a new English version. A monolingual reviewer on the research team then compared the original and back translated versions of the diagnostic interview guide questionnaire. The initial question inquired whether the patient had experienced significant fatigue, a lack of energy, or an increased need to rest for a least a 2-week period during the last month. Given the many causes of fatigue and its multidimensional manifestations, the next 10 questions included 10 common clinical manifestations of fatigue reported by cancer patients. The last 3 questions were arranged to exclude non-CRF (Table 2).

**Table 2:** Diagnostic interview guide for cancer-related fatigue

Diagnostic Interview Guide for Cancer-Related Fatigue			
**NOTE**: Capitalized text represents instructions to the interviewer. Text in quotations represents statements to be read verbatim to respondent.			
1 "Over the past month, has there been at least a 2-week period when you had significant fatigue, a lack of energy, or an increased need to rest every day or nearly every day?"	CIRCLE ONE:	**YES**	**NO**
**IF NO, STOP HERE. IF YES, CONTINUE**			
"For each of the following questions, focus on the worst 2 weeks in the past month (or else tha past 2 weeks if you felt equally fatigued for the entire month)."			
2. "Did you feel weak all over or heavy all over? (every day or nearly every day?)"	CIRCLE ONE:	**YES**	**NO**
3. "Did you have trouble concentrating or paying attention? (every day or nearly every day?)"	CIRCLE ONE:	**YES**	**NO**
4. "What about losting your interest or desire to the things you usually do? (every day or nearly every day?)"	CIRCLE ONE:	**YES**	**NO**
5. "How were you sleeping? Did you have trouble falling asleep, staying asleep, or waking too early? Or did you find yourself sleeping too much compared to what you usually sleep? (every day or nearly every day?)"	CIRCLE ONE:	**YES**	**NO**
6. "Have you found that you usually don't feel rested or refreshed after you have slept? (every day or nearly every day?)"	CIRCLE ONE:	**YES**	**NO**
7. "Did you have struggle or push yourself to do anything? (every day or nearly every day?)"	CIRCLE ONE:	**YES**	**NO**
8. "Did you find yourself feeling sad, frustrated, or irritable because you felt fatigued? (every day or nearly every day?)"	CIRCLE ONE:	**YES**	**NO**
9. "Did you have difficulty finishing something you had started to do because of feeling fatigued? (every day or nearly every day?)"	CIRCLE ONE:	**YES**	**NO**
10. "Did you have trouble remembering things? For example, did you have trouble remembering where your keys were or what someone had told you a little while ago? (every day or nearly every day?)"	CIRCLE ONE:	**YES**	**NO**
11. "Did you find yourself feeling sick or unwell for several hours after you had done something that took some effort(every time or nearly every time?)"	CIRCLE ONE:	**YES**	**NO**
**IF LESS THAN SIX ITEMS INCLUDING #1 ARE MARKED YES, STOP HERE**.			
12. "Has fatigue made it hard for you to do your work, take care of things at home, or get along with other people?"	CIRCLE ONE:	**YES**	**NO**
**IF #12 IS NO, STOP HERE**			
13. IS THERE EVIDENCE FROM THE HISTORY, PHYSICAL EXAMINATION, OR LABORATORY FINDINGS THAT THE SYMPTOMS ARE A CONSEQUENCE OF CANCER OR CANCER TREATMENT?	CIRCLE ONE:	**YES**	**NO**
**IF #13 IS NO, STOP HERE**			
14. ARE THE SYMPTOMS PRIMARILY A CONSEQUENCE OF CO-MORBID PSYCHIATRIC DISORDERS SUCH AS MAJOR DEPRESSION, SOMATIZATION DISORDER, SOMATOFORM DISORDER, OR DELIRIUM?	CIRCLE ONE:	**YES**	**NO**
**IF #14 IS YES, PATIENT DOES NOT MEET CRITERIA FOR CANCER-RELATED FATIGUE**			
**IF #14 IS NO, PATIENT MEETS CRITERIA FOR CANCER-RELATED FATIGUE**			

Prior to the data collection for the current study, the coordinator of this study (Y.L.) met the interviewers of this study (E.Y, Y.T., and W.S.) for training purposes. The training involved a review of the diagnostic criteria, the completion of simulated interviews, and the completion of actual interviews conducted with patients who were not participants of this study. The interviews of this study were conducted by 2 physicians and 1 nurse who was also currently a graduate student. They were all experienced in working with cancer patients. Sixteen interviews were tape-recorded for the purpose of determining inter-rater reliability. Ratings obtained from the interviewers were compared with those from the independent rater (S.L.) who was trained for the administration of the interviews but not involved in the data collection of this study. The level of the overall agreement between the study interviewers and the independent rater regarding the presence/absence of cancer-related fatigue was 95%. After calculation, we obtained that the coefficient of kappa, a measurement adjusted for base rates, was 0.88. The level of the overall agreement between the study interviewers and the independent rater regarding the presence/absence of the 14 individual criteria that comprise the diagnostic interview guide for Cancer-Related Fatigue (kappa = 0.96) was 90%. According to the rule of thumb, values of Kappa from 0.40 to 0.59 are considered moderate, 0.60 to 0.79 substantial, and 0.80 outstanding (Landis & Kochi, 1977). Most statisticians prefer Kappa values to be at least 0.6 and most often higher than 0.7 before claiming a good level of agreement.

A pretest was performed in which 21 Chinese-language versions of the diagnostic interview guide were completed before the study. Descriptive statistics were computed per patient group as P-ICD-positive or P-ICD-negative. Internal consistency of the P-ICD-10 was assessed by computing Cronbach's alpha coefficient. The correlations of each question were examined, and the values of the alpha coefficient showed medium strength correlations (ranging from 0.11 to 0.3). These indicated the instrument is a reliable tool for measuring fatigue.

## Results

### Participant Characteristics

Demographic and disease-related characteristics of the patients are presented in Table 3. Data on the 265 patients, of whom 64% were women, were collected between 21 October 2008 and 28 October 2009 at two Taipei clinical centers (Mackay Memorial Hospital, n = 200, Cardinal Tien Hospital, n = 65). Of the 265 patients, 228 (86%) answered "yes" to the first question of the questionnaire, which corresponds to criterion A1 of the P-ICD criteria of CRF, and requires a person to report a minimum of 2 weeks of fatigue in the past month. Criterion A specifies that six or more symptoms must have been resent every day or nearly every day during the same 2-week period of time over the past month and at least one symptom must be significant fatigue. Table 4 lists individually by symptom, the number and the percentages from criteria A1 to A11, which corresponds to questions number 1 to 11, respectively. Criterion B specifies that the fatigue symptoms must cause clinically significant distress or impairment in social, occupational, or other important areas of functioning. Thirty patients (11%) had six or more of the symptoms in criterion A but responded that the symptoms did not cause significant distress or impairment in social, occupational, or other important areas of functioning (Table 5). The part in Table 4 in bold fonts (A1 + any 5 + B) shows that criteria A and B of the P-ICD-10 diagnostic criteria were met by 28 (11%) patients. This result is compared with a western study of 375 patients conducted by Cella D, Davis K, Breitbart W, et al. showing that 17% of patient respondents met both Criteria A and B [11]. In addition, of the 265 patients, 132 (49.8%) were classified as fatigue-positive according to the P-ICD-10. 66 subjects were more than 70 years old (24.9%), 61 subjects were between 61 to 70 years old (23%), 81 subjects were between 51 to 60 years old (30.5%), 37 subjects were between 41 to 50 years old (13.9%), 14 subjects were between 31 to 40 years old (5.2%), and 3 subjects were below 30 years old (1/1%). 15% of the patients did not receive education, and 22% had some college or higher education. The different age groups and education levels were not statistically significant if they were classified as P-ICD 10-positive and P-ICD 10-negative. The participants were diagnosed with various types of cancer, including head and neck (7.9%), lung and mediastinum (10.5%), breast (36.6%), liver (3.7%), GI tract (16.6%), GU tract (3.3%), GYN (6.4%), prostate (3.3%), hematological malignancy (0.3%), and others (8.6%). Notably, the breast (p = 0.033) and GI tract (0.035) were statistically significant if they were classified as P-ICD 10-positie and P-ICD 10-negative (p < 0.05). Among the 132 patients who were classified as fatigue-positive according to the P-ICD-10, 77 patients are under palliative treatment, 18 patients received curative treatment, and 35 patientswere follow up after curative therapy. The group under palliative treatment and curative treatment were statistically significant if they were classified as P-ICD 10-positive and P-ICD 10-negative (p < 0.05). In addition, regarding the staging of the disease, the group under no signs and symptoms and tumor progression were statistically significant if they were classified as P-ICD 10-positive and P-ICD 10-negative (p < 0.005).

**Table 3:** Sample Characteristics

	P-ICD 10-positive(N = 132) No. (%)	P-ICD 10-negative(N = 133) No. (%)	P-value
Sex			
Male	49 (37)	44 (33)	
Female	83 (63)	89 (67)	
Age			
> 70	36 (27)	30 (23)	
61-70	33 (25)	28 (21)	
51-60	37 (28)	44 (33)	
41-50	16 (12)	21 (16)	
31-40	8 (6)	6 (5)	
< 30	1 (1)	2 (2)	
Education			
Did not receive education	24 (18)	16 (12)	
Elementary	42 (32)	43 (32)	
high school	36 (27)	38 (29)	
College	25 (19)	29 (22)	
College graduate	1 (1)	4 (3)	
Missing	4	3	
Cancer diagnosis			
Head & Neck	9	12	
Lung & Mediastinum	12	16	
Breast	39	58	0.033*
Liver	5	5	
GI tract(except liver)	29	15	0.035*
GU tract	6	3	
GYN	8	9	
Prostate	7	2	
Hematological malignancy	2	0	
Others	10	13	
Missing	2	3	
Treatment Modalities			
Palliative treatment	77	51	0.022*
Curative treatment	18	38	0.088*
F/U after curative treatment	35	43	
Condition of the Disease	23	0	
No signs and symptoms	30	51	0.02*
Stationary state	13	20	
Progression (local invasion)	35	18	0.02*
Progression (Metastasis)	28	15	0.047*
Terminal stage	27	21	
Missing	5	2	

**Table 4:** Patients who endorsed category A criteria (N = 265)

Criterias	N	%
A1 Two weeks of fatigue in past month	228	86%
A2 General weakness	172	65%
A3 Trouble concentrating	133	50%
A4 Decreased motivation	115	43%
A5 Insomnia/hypersomnia	162	61%
A6 Nonrestorative sleep	175	66%
A7 Having to push things	113	43%
A8 Sadness or frustration	106	40%
A9 Trouble completing daily tasks	125	47%
A10 Short-term memory problems	162	61%
A11 Postexertional malaise	123	46%

**Table 5:** Patients who endorsed multiple symptoms (Criteria A) and Criteria B (N = 265)

Multiple Symptoms Reports	N	%
Did not responded to A1	37	14%
Only responded A2	4	2%
A1 + any 1	7	3%
A1 + any 2	12	5%
A1 + any 3	26	10%
A1 + any 4	20	8%
A1 + any 5 + B*	28	11%
A1 + any 6 + B	14	5%
A1 + any 7 + B	21	8%
A1 + any 8 + B	23	8%
A1 + any 9 + B	22	8%
A1 + any 10 + B	24	9%
A1 + any 5 or more but does not responded to B	30	11%

### Internal consistency of the P-ICD-10

Occurrences of each P-ICD-10 fatigue symptom in all patients, and patients classified as P-ICD 10-positive (N = 129) and P-ICD 10-negative (N = 380) are listed in Table 6.

**Table 6:** Occurrences of each P-ICD 10 symptoms classified as positive and negative

	P-ICD 10 positive N (%)	P-ICD 10 negative N (%)
A2 General weakness	113 (87.6%)	59(43.4%)
A3 Trouble concentrating	87(67.4%)	46(33.8%)
A4 Decreased motivation	85(65.9%)	30(22.1%)
A5 Insomnia/hypersomnia	101(78.3%)	61(44.9%)
A6 Nonrestorative sleep	114(88.4%)	61(44.9%)
A7 Having to push things	91(70.5%)	22(16.2%)
A8 Sadness or frustration	81(62.8%)	25(18.4%)
A9 Trouble completing daily tasks	98(76.0%)	27(19.9%)
A10 Short-term memory problems	105(81.4%)	57(42.0%
A11 Post-exertional malaise	94(72.9%)	29(21.3%)

According to the design of the questionnaire, the 1^st ^question, which corresponds to the A1 criteria of the P-ICD-10 criteria, should be answered "yes" in order for the questionnaire to proceed, thereby resulting in the non-discriminating property of this general symptom. Of the 10 other complaints, the most frequently reported by both P-ICD 10-positive and P-ICD 10-negative patients were "general weakness", "insomnia or hypersomnia", "experience of sleep as unrefreshing or non-restorative", and "perceived problems with short-term memory". The most discriminative complaints were related to physical fatigue, and they are listed according to the order of their discriminative properties: "Difficulty completing daily tasks attributed to feeling fatigued" (P-ICD 10-positive 76%, negative 19.9%), "Perceived need to struggle to overcome inactivity" (P-ICD 10-positive 70.5%, negative 16.2%), and "Post-exertional malaise lasting several hours" (P-ICD 10-positive 72.9%, negative 21.3%). The least discriminating of the complaints was about sleep quality, which is the 5^th ^question on the questionnaire: "insomnia or hypersonmia".

Internal consistency was established by calculating Cronbach alpha coefficients, which were 0.843, indicating good internal consistency for the Chinese-language version of the P-ICD 10 criteria. Removing non-reliable items would increase internal consistency. The alpha coefficient did not increase after removing any individual item, removing any item decreased the internal consistency, and the most reliable items, aside from the 1^st ^symptom, were the 7^th^, 9^th^, and 2^nd ^symptom, respectively.

### Content Validity

To guarantee cultural equivalence and to establish expert validity, 7 authoritative specialists in the field of nursing, oncology, radio-oncology, psychiatry, and linguistics were formed to examine the Chinese-language version of the P-ICD 10 criteria. The experts were asked to rate the relevance of the content independently using a content validity index (CVI). They were asked to rate each item on the Chinese-language versions of the diagnostic interview guide based on relevance and semantic equivalence using the following 4-point Likert scale: 1 = not relevant (not appropriate), 2 = somewhat relevant (somewhat appropriate), 3 = relevant (quite appropriate), 4 = very relevant (very appropriate). The CVI was then computed based on the percentage of total items rated by the experts as either 3 or 4. According the Norwood(2000), a CVI rating exceeding .80 can be considered to show good content validity. All items in the Chinese-language versions of the diagnostic interview guide were rated by the panel experts as having a CVI greater than .90. For the final version, the CVIs for relevance and semantic equivalence both reached .99.

## Discussion

Fatigue is an important and one of the most prevalent complaints of cancer patients both during and after treatment [13], and previous reports demonstrated that the prevalence of cancer fatigue is up to 90% [14]. In this study, 86% of patients reported significant fatigue, which is the first question of the questionnaire, indicating that it is a very common symptom for Taiwanese cancer patients. This result is consistent with other studies. The prevalence of CRF was studied in 2 national surveys conducted by Fatigue Coalitions, which studied 419 and 197 cancer patients, and showed an overall 74% of patients reporting fatigue [15]. Another large-scale survey of 379 cancer patients who had received chemotherapy with or without radiation therapy showed fatigue was present in 75% of patients, with patients reporting fatigue as the symptom that most affected QOL, followed by nausea, depression, and pain [16]. In another study, 58% of a sample of cancer patients receiving cancer therapy reported that fatigue had affected them in the past month, and that fatigue affected them significantly more than any other cancer symptom [6]. In addition, another study reported that up to 75% of advanced-cancer patients suffered from significant fatigue [17]. These results indicated that fatigue is a prevalent symptom related to cancer and cancer therapy. However, cancer fatigue is often underestimated as the oncologist's attention is focused primarily on tumor-related parameters such as disease-free survival or on treatable symptoms such as pain [18]. This situation is explained by a lack of communication between physician and patient regarding fatigue [15]. The lack of interest in fatigue as a topic for research has also been attributed to the lack of any effective treatment strategy [19]. A survey of 470 health care personnel from various clinical areas demonstrated their poor knowledge and practice regarding fatigue assessment and management [20].

Another reason for the lack of interest in research on CRF is the lack of validated and clinically easy-to-use assessment tools [13]. Similar to pain, fatigue is commonly conceptualized as a multidimensional sensation that incorporates sensory, cognitive, affective, behavioral, and physiologic components [21]. However, unlike pain, no universally accepted definition or well-conceptualized dimensions for fatigue have been proposed [22]. Although a multidimensional measurement may seem appropriate, and there are currently numerous multidimensional scales available, those scales may be too long for fatigued patients to complete and are therefore not suitable for clinical use. Moreover, unidimensional scales such as the BFI may not capture the multiple dimensions of fatigue and therefore are more suitable for screening or measuring the intensity of fatigue. This picture supports the need for reliable instruments to diagnose and assess cancer-related fatigue. Since the P-ICD-10 criteria have good internal consistency and can be recommended as a diagnostic tool [12], they were used with 265 patients in 2 clinical centers in Taiwan. The P-ICD-10 is structured as a multidimensional instrument and the present results confirm its good internal consistency with an alpha coefficient of 0.843 with all individual items being reliable. "Insomnia or hypersomnia", showed the lowest correlation with the other items, though it is also one of the most frequently reported symptoms of both ICD-10-positive and ICD-10-negative symptoms.

Although 86% of our patients reported fatigue, further investigation using the P-ICD-10 criteria demonstrated a significantly lower percentage (48.6%), as compared with currently reported estimates of CRF, which range from 60% to 90%. However, unlike all other previous studies that provide the percentage of individuals who reported any degree of fatigue, regardless of its impact on functioning, this study required that there be significant fatigue-related problems and disruption in daily functioning to assign the diagnosis. Thus, we distinguished between fatigue as a symptom reported by the vast majority of people with cancer, and CRF as a diagnostic entity.

The most frequently reported symptom of our sample population, whether the patients were classified as having CRF or not, was that of the 6^th ^question in the questionnaire, which corresponds to criteria A6 of the P-ICD-10, "experience of sleep as unrefreshing or non-restorative", and it was also the symptom most frequently reported by both the P-ICD 10 positive and negative patients. Sleep disturbance associated with fatigue is often difficult to treat and manage. It may be influenced by numerous factors, including daytime naps, depression, anxiety, certain medications, tumor pain, sleep disruption on account of increased urination or hot flushes, and evening food and beverage intake. Although sleep disturbance is common in patients with cancer, it has been evaluated in few studies. In a pilot study of women receiving adjuvant chemotherapy for breast cancer, stimulus control (i.e., having a consistent time to lie down and get up, avoiding caffeine and stimulating activity in the evening) and sleep restriction (i.e., avoiding long or late afternoon naps, limiting time in bed to the normal hours of sleep only) significantly improved fatigue [23]. The benefit of a multimodality sleep hygiene program was suggested in a controlled study of 2 different interventions (relaxation techniques or autogenic training) for up to 6 months; compared with the control group, both intervention groups had significantly better sleep latency, sleep duration, and daytime functioning [24]. In addition, cognitive-behavioral therapy and stress reduction also may help insomnia and sleep disorder [25].

The 2^nd ^most frequently reported symptom among our sample patients, whether they were diagnosed as having CRF or not, was that of the 2^nd ^question on the questionnaire, "complaints of generalized weakness or limb heaviness". With an incidence of 65%, which was related to physical fatigue, it was also the 2^nd ^most frequently reported symptom by both the P-ICD-10-positive and negative patients. In addition, the most discriminating complaints were also related to physical fatigue, such as "difficulty completing daily tasks attributed to feeling fatigue", "perceived need to struggle to overcome inactivity", and "post-exertional malaise lasting several hours". This indicated that physical issues are important factors in fatigue among cancer patients, and the incidence is even higher for patients who met the P-ICD-10 criteria. Quite a number of previous studies identified a decrease in activity level and muscle power related to cancer patients [26]. A consistent decrease in the amount of daily activity could eventually lead to a reduced tolerance for normal activity and result in high levels of fatigue. The specific mechanism causing such a phenomenon remains essentially unknown. Current theory links cancer asthenia with a similar mechanism purported to play a role in producing the anorexia-cachexia syndrome, and the profound fatigue and fever associated with some infectious diseases. An immune response to the tumor resulting in activation of macrophages and the release of cytokines, such as tumor necrosis factor, IL-1, IL-6, and interferon-r, is central to the current model, as is the release of tumor by-products [27,28]. Exercise has been shown to have the strongest evidence of benefit [29]. A structured aerobic exercise program has been shown in several trials to reduce fatigue and emotional distress and to improve quality of life in cancer patients who exercised during treatment. Exercise regimens usually included walking or cycling [30-32]. According to NCCN guidelines for the management of CRF, exercise is the intervention with the most supporting evidence for effectiveness (category 1).

Fatigue is a multidimensional symptom. It may lead to influences on physical, psychological, spiritual and the social field. However, even now, the cause of fatigue cannot be readily identified, and it is difficult to properly diagnose the symptom. Thus there is no specific treatment for fatigue. And possible influential factors cannot be removed to ease the symptom and show curative effect. We could only reduce the symptom and provide the psychosocial and spiritual support for those with CRF. For the patients with fatigue, we recruit them for the holistic approach of palliative care which includes psychosocial and spiritual approach and psycho-oncology consultation. As for those with progressive fatigue, trials with Steroids and Ritalin were applied with some subjective response. The symptom management also includes non-pharmacological intervention, such as exercise, stress reduction, counseling, and psychosocial support. Complementary and alternative medicine such as Chinese Medicine might play some roles in fatigue management and should be studied in the future. The development of guidelines for managing fatigue in cancer patients depends on continued progress in understanding its pathophysiology and many contributing factors, and we believe the treatment of fatigue will be optimized by proper diagnosis, and a comprehensive strategy will probably produce the best results given the many causes of fatigue and its multidimensional manifestation.

The results of this study should be interpreted with caution because of certain limitations. There is a possibility that the sample of patients studied is not representative of the general population of Taiwan cancer patients, since the patients recruited were from northern Taiwan. Moreover, patients with cognitive impairment and poor communication skills were excluded, and this may result in underestimation of the prevalence of fatigue.

## Conclusion

In conclusion, the P-ICD-10 criteria for CRF have good internal consistency, and are a reliable and clinically easy-to-use measurement of fatigue in Taiwanese cancer patients. In addition, our results showed a high incidence of fatigue among our sample of cancer patients, mostly due to sleep disturbances and physical factors. Further investigation of the management of fatigue, including pharmaceutical, psychological or physical treatment, is greatly needed. It is our hope that the refining and use of these diagnostic criteria will help better support CRF research and clinical management, and improve communication between patients and clinicians.

## Competing interests

The authors declare that they have no competing interests.

## Authors' contributions

EY conceived the study and drafted the manuscript. SL assisted in drafting of the manuscript and provided critical revision. DT aided in the design of the study and provided critical revision of the manuscript. YT assisted in the design of the study and aided in data collection. WS carried out the analysis and aided in data collection. YL led the study design and data collection and coordinated the drafting of the manuscript while providing critical revision. All authors read and approved the final manuscript.

## Pre-publication history

The pre-publication history for this paper can be accessed here:

http://www.biomedcentral.com/1471-2407/11/387/prepub
